# Pregnancy Induced Hypertensive Disorders among Patients Admitted to the Department of Obstetric and Gynecology in a Tertiary Care Centre: A Descriptive Cross-sectional Study

**DOI:** 10.31729/jnma.8060

**Published:** 2023-03-31

**Authors:** Sushma Lama, Padma Gurung, Anagha Pradhan Malla

**Affiliations:** 1 Department of Obstetrics and Gynaecology, Patan Academy of Health Sciences, Lagankhel, Kathmandu, Nepal

**Keywords:** *preeclampsia*, *pregnancy induced hypertension*, *prevalence*

## Abstract

**Introduction:**

Hypertensive disorder of pregnancy is among the leading causes of maternal and perinatal mortality in developing countries. There are only few studies regarding this topic so this study helps us to improve our management protocol thereby reducing maternal and foetal morbidity and mortality. The aim of this study was to find out the prevalence of pregnancy induced hypertensive disorder among patient admitted to the Department of Obstertric and Gynecology in a tertiary care centre.

**Methods:**

A descriptive cross sectional study was conducted in the Department of Obstetrics and Gynaecology of tertiary care centre from 30 July 2020 to 30 July 2021 after obtaining ethical approval from the Institutional Review Committee (Reference number: 2007211399). Convenience sampling method was used among patients who met the eligibility criteria. Point estimate and 95% Confidence Interval were calculated.

**Results:**

Among 4,303 deliveries, hypertensive disorder in pregnancy was seen in 110 (2.55%) (2.083.03, 95% Confidence Interval) Among 110 (2.55%) patients, preeclampsia was seen in the majority of the patients 69 (62.72%).

**Conclusions:**

The prevalence of hypertensive disorder among pregnancies was similar to the w studies done in similar settings. Hypertensive disorder poses a major issue in pregnant women so should be taken into a serious matter as it causes major problems in maternal and foetal outcomes.

## INTRODUCTION

Hypertensive disorder of pregnancy are among the leading cause of maternal and perinatal mortality in developing countries.^1^ Hypertension affects more than 5-8% of all pregnancies in the world.^2^ Maternal complications includes acute renal failure, hepatic failure, postpartum haemorrhage, disseminated intravascular coagulation, abruptio, and cerebrovascular accident. Foetal complications includes premature deliveries, intrauterine growth restriction, stillbirth, and neonatal deaths.^2,3^

Although there are some tests for prediction or early detection of preeclampsia like uterine artery doppler and maternal serum markers, there is not enough evidence to suggest their use in clinical practice more so in poor resource clinical settings.^4-6^ In Nepal, there are only few studies regarding this topic so this study helps us to improve our management protocol thereby reducing maternal and foetal morbidity and mortality.

The aim of this study was to find out the prevalence of pregnancy induced hypertensive disorders among patients admitted to the Department of Obstetrics and Gynaecology in a tertiary care centre.

## METHODS

This descriptive cross sectional study was done in the Department of Obstetrics and Gynaecology of a Patan Academy of Health Sciences (PAHS). The duration of study was one year from 30 July 2020 to 30 July 2021. Ethical approval for the study was taken from the Institutional Review Committee of the PAHS (Reference number: 2007211399). All pregnant women admitted in the maternity ward within the study period were included in the study. Patients with chronic hypertension (when hypertension begins before 20 weeks of gestation or exists before pregnancy), chronic hypertension with superimposed preeclampsia and those with pre-existing medical conditions like renal, vascular, systemic lupus erythematosus, thyrotoxicosis were excluded from the study. Convenience sampling method was used. The sample size was calculated using the formula:


$$\begin{array}{ll} \text{n} & ={\text{Z}}^{2}\times \frac{\text{p}\times \text{q}}{{\text{e}}^{\text{2}}}\\ & =1.96^{2}\times \frac{0.50\times 0.50}{0.02^{2}}\\ & =1068 \end{array}$$

Where,

n = minimum required sample size
Z = 1.96 at 95% Confidence interval (CI)
p = prevalence taken as 50% for maximum sample size calculation
q = 1-p
e = margin of error, 2%

The minimum sample size obtained was 2401. Since convenience sampling was used, the sample size was quadrupled which was 4,272. However, 4303 patients were included in the study.

According to the American College of Obstetrics and Gynaecology, pregnancy induced hypertension is divided into four groups: gestational hypertension where blood pressure is 140/90 mm Hg or more after 20^th^ week of gestation without proteinuria, preeclampsia is raised BP with proteinuria, chronic hypertension is that exists before pregnancy or begins in the first 20 weeks of gestation, preeclampsia superimposed on chronic hypertension (chronic hypertension with proteinuria) and eclampsia is preeclampsia with seizures.^7^

Data were collected from patient's files regarding maternal age, parity, gestational age, type of hypertensive disorder, type of delivery, maternal complications, and foetal birth weight, preterm, intrauterine foetal death, neonatal death, neonatal intensive care unit, and nursery admission were noted.

Data were entered and analysed by using Microsoft Excel 2016. Point estimate and 95% Confidence Interval were calculated.

## RESULTS

Among 4,303 women, who delivered in our hospital during the study period, the prevalence of hypertensive disorder in pregnancy was 110 (2.55%) (2.08-3.03, 95% CI). In this study, 69 (62.72%) of the patients had preeclampsia, 34 (30.90%) had gestational hypertension and 7 (6.36%) had eclampsia. Among eclampsia, 5 (71.43%) patients had antepartum eclampsia and 2 (28.57%) had postpartum eclampsia (Table 1).

**Table 1:** Distribution on types of hypertensive disorder and outcome (n= 110).

Parameters	n (%)
Types of hypertensive disorder	
Gestational hypertension	34 (30.90)
Preeclampsia	69 (62.72)
Eclampsia	7 (6.36)

Out of 110 patients with hypertensive disorders of pregnancy 78 (70.90%) patients had caesarean section among which 60 (54.54%) were elective and 18 (16.36%) were emergency caesarean section. There were a total 32 (29.09%) vaginal deliveries.

There were 2 (0.90%) patients who had acute renal failure, 1 (50%) of the patients had six cycles of haemodialysis. There were 4 (3.63%) postpartum haemorrhage in which 3 (75%) were managed medically and also received blood products and in 1 (25%) patient bilateral uterine artery ligation was done and condom tamponade was also inserted.

There was 1 (0.90%) re-laparotomy following hemoperitoneum. A total of 9 (8.18%) patients were COVID-19 positive. There were 2 (1.81%) mortality, in which first died due to disseminated intravascular coagulation, acute renal failure and pulmonary oedema, second one died due to acute respiratory distress syndrome and pulmonary oedema. Both the patients also had COVID-19 pneumonia. Both died in the postpartum period and both were intubated. One expired on day 7 and the other on day 11 (Table 2).

**Table 2:** Mode of delivery and maternal complications (n= 110).

Parameters	n (%)
Mode of delivery	
Vaginal	32 (29.09)
Caesarean section	78 (70.90)
Maternal complications	
Oligohydramnios	7 (6.36)
HELLP	6 (5.40)
Abruptio	6 (5.40)
Postpartum haemorrhage	4 (3.63)
Acute renal failure	2 (1.81)
PRES(Posterior reversible encephalopathy syndrome)	2 (1.81)
Hypertensive retinopathy	2 (1.81)
Retinal haemorrhage	2 (1.81)
Mortality	2 (1.81)
Disseminated intravascular coagulation	1 (0.90)
Acute respiratory distress syndrome	1 (0.90)
Pleural effusion	1(0.90)
Pericardial effusion	1 (0.90)
Pulmonary oedema	1 (0.90)
Re-laparotomy	1 (0.90)

There were 17 (15.45%) babies who were <1.5 kg. In complication, the majority of the cases were intrauterine growth restriction 49 (44.54%) and prematurity 40 (36.36%). There were 9 (8.18%) intrauterine foetal death (Table 3).

**Table 3:** Foetal outcome and complications (n= 110).

Parameters	n (%)
Birth weight	
< 1.5	17 (15.45)
1.5-2.4	37 (33.63)
2.5-3.5	40 (36.36)
>3.5	16 (14.54)
Complications	
Preterm	40 (36.36)
Intrauterine growth restriction 49 (44.54)	
Intrauterine foetal death	9 (8.18)
Post delivery	
Neonatal intensive care unit	11(10)
Nursery	5 (4.54)
Neonatal death	5 (5.54)

The mean±SD of age was 29.48±4.95 years varying from a minimum of 19 years to maximum of 35 years. There were 55 (50%) patients in either group; primigravida and multigravida group. The mean±SD of gestational age was 35.66±3.47 weeks (minimum= 20 weeks and maximum= 40 weeks) (Table 4).

**Table 4:** Distribution of age, parity and gestational age (n= 110).

Parameters	n (%)
Age group in years	
<20	3 (2.72)
20-25	21 (19.09)
26-29	24 (21.81)
30-35	53 (48.18)
>35	9 (8.18)
Gravida	
Primigravida	55 (50)
Multigravida	55 (50)
Gestational age	
<28	3 (2.72)
28-33	18 (16.36)
34-36	19 (17.27)
≥37	70 (63.63)

## DISCUSSION

Hypertensive disorders in pregnancy are considered a major problem affecting both maternal and foetal morbidity and mortality. The prevalence of hypertensive disorder in pregnancy differs worldwide. In Sweden it is 1.5%, 7.5% in Brazil and 6-8% in India.^7^ Out of total 4,303 deliveries, 110 deliveries were found to be complicated by hypertensive disorder in pregnancy hence the prevalence was 2.55% in our study.

The extremes of age are well known risk factors for hypertension in pregnancy. In our study most of the participants were in the age group of 30 to 35 (48.18%) where as a previously studied showed, the participants were in the age group of 21 to 25 years,^8^ and 20 to 30.^9^ Another study found that the age above 30 years were associated with risk for preeclampsia.^10^ In our study number of primigravida and multigravida was equal 50% whereas in previously study showed 60.8% was primigravida and 39.2% multigravida.^11^ Primigravida has 15 times greater risk for developing preeclampsia as compared to multigravida.^12^

The most common maternal complications were HELLP, abruptio and oligohydramnios in our study. Similar study also showed HELLP syndrome as common complication followed by abruptio, acute renal failure and postpartum haemorrhage.^11^ Another study found a significant association between the occurrence of HELLP syndrome and maternal mortality and morbidity.^13^ In our study 5.4% patients had HELLP syndrome. In our study there were 2 (0.90%) maternal mortalities, in which first died due to disseminated intravascular coagulation, acute renal failure and pulmonary oedema, second one died due to acute respiratory distress syndrome and pulmonary oedema. Both the patients were also COVID-19 positive. Whereas as in a previous study also showed had two mortality which were due to intracranial haemorrhage.^14^

A study showed the majority of the patients had eclampsia 40.6% whereas in our study most of the patients presented with features of preeclampsia.^9^ There were only seven cases of eclampsia in our study. Similar study showed majority of patients had preeclampsia (59.6%).^11^ In another study showed gestational hypertension was most common (65.62%), followed by preeclampsia (28.12%) and eclampsia (6.25%).^15^ In our study there were 70.90% caesarean section out of which 54.54% were emergency and 18% were elective and 29.09% were vaginal delivery. Similar study showed out of 123 patients 105 (85.2%) had caesarean section,^17^ had vaginal delivery and 1 had vaginal birth after caesarean section.^9^ whereas in another study 63% had vaginal delivery and 37% had caesarean section.^16^

The risk of prematurity with PIH is approximately 25-30%.^17^ A study showed IUGR and prematurity as the commonest foetal complications which was similar to our study.^18^ In our study 36.36% of the pregnancies ended up in preterm deliveries, majority of which occurred due to maternal and foetal indication for termination of pregnancy. The definite treatment of preeclampsia is termination of pregnancy, which is done despite of risk of prematurity irrespective of the gestational age to avoid maternal complications and morbidity.^2^ Another study showed, out of 64 deliveries 18.75% babies required NICU admission for various causes with 1.56% IUFD and 1.56% neonatal death. 19 However our study showed 10% NICU admission, 8.18% IUFD and 4.54% neonatal death.

There are some limitations in our study. Since preeclampsia is influenced by race, parity, ethnicity, environmental factors, socio-economic status, obesity, these parameters have not been included in our study so the association could not be explored. A multicentre based larger population study might have a different outcome compared to that of this study.

## CONCLUSIONS

The prevalence of hypertensive disorder among pregnancies was similar to the other studies done in similar settings. Emphasis should be on early registration, regular antenatal visits, identifying the high risk group, starting medication on time, timely referral to higher centres which can prevent and reduce severity and its associated complications. Awareness about hypertension should not just be spread from the hospital but should be from the community level as well. Women should be educated about regular blood pressure monitoring and to do routine urinary protein analysis at every antenatal visit.
